# Fusion of the Median and Musculocutaneous Nerves Masquerading as Biceps' Innervation from the Median Nerve

**DOI:** 10.1055/s-0043-1767674

**Published:** 2023-11-15

**Authors:** Ioannis Antonopoulos, Margarita- Michaela Ampadiotaki, George Tsikouris, Ioannis Chiotis, Georgios Tsakotos, Ioannis Pathiakis, Theodore G. Troupis

**Affiliations:** 1 Department of Anatomy, School of Medicine, National and Kapodistrian University of Athens, Zografou, Greece

**Keywords:** median nerve, musculocutaneous nerve, biceps' innervation, brachial plexus

## Abstract

Embryologically, the musculocutaneous nerve (MCN) comes from the lateral root of the median nerve, and thus numerous anatomical variations concerning the formation and branching pattern of these two nerves of the brachial plexus have been described. In this case study, we describe a relatively uncommon case of fusion of the median and MCNs that was identified during routine teaching dissection of a male human cadaver. The identification of this anatomical variation requires awareness of the embryological background, as it may be confused with biceps innervation from the median nerve or the existence of a communicating branch between the two nerves. In addition, awareness of such anatomical variations is of undisputable significance for the safety of surgical operations in the brachial plexus and the arm in general.

## Introduction

The brachial plexus (BP) is formed by the contribution of all the anterior divisions of the C5 to T1 nerves and a small contributing branch of the fourth cervical nerve (C4). These nerves join to form the three trunks and three cords of the BP (and finally the nerves that arise from it) in a way that significantly varies. The musculocutaneous nerve ( MCN, C5–C7) comes from the lateral cord of the BP, whereas the median nerve (MN, C5–T1) from both the lateral and the median cord, formed by two neural roots that join in a “V”-shaped configuration. 1 This MN formation may also vary as there have been described cases of single-root or four-roots formation or even bifid or trifid roots. 2 3 4 As for the innervation of the brachial muscles it is known that all the three muscles of the anterior compartment of the arm are innervated by the MCN and the posterior ones by the radial nerve. 1

The anatomy of the BP is quite complex, mainly due to its numerous variations, especially those including the existence of communicating branches between BP nerves, for instance between the MN and MCN or the ulnar and radial nerves. 5 More specifically the variations of the MCN are quite common as it may pass beneath or even through the biceps rather than perforating the coracobrachialis muscle (CBM), as well as it may send a communicating branch to the MN. In this case study, we aim to describe in detail a relatively rare case of partial fusion of the MN and the MCN and highlight its potential clinical significance.

## Case Report

The reported case was identified during the dissection of the right axilla and arm of a male formalin-fixed (10% v/v solution) cadaver. The dissection was held for both educational and research reasons at the dissection hall of our anatomy department. The cadaver was of Greek origin and derived from body donation with the written and informed consent of the donor, according to the relevant legislation. 6 The specimen was properly cleaned and photographed. A Würth digital Vernier caliper (0.01 mm, accuracy) was used for the measurements of the distances and nerves' diameters.

**Fig. 1:**
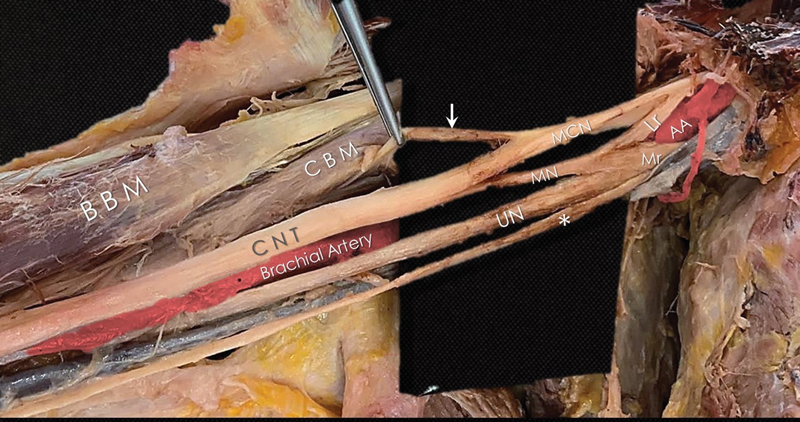
The formation of the terminal branches of the brachial plexus from the medial and lateral cords. Muscular to the coracobrachialis muscle ( *white arrow* ). AA, axillary artery; Lr, lateral root of the median nerve; BBM, biceps brachialis muscle; CBM, coracobrachialis muscle; CNT, common neural trunk; MCN, musculocutaneous nerve; MN, median nerve; Mr, medial root of the median nerve; UN, ulnar nerve; (*), medial cutaneous brachii nerve.

As depicted in Fig. 1 the MN (diameter—d: 1.10 mm) was formed, as usual, from a lateral and a medial root at the level of the border between the second and third segments of the axillary artery. We identified the cords and the terminal branches of the BP, and we first thought that we were facing a case of a communicating branch from the MCN (d: 1.98 mm) to the MN. However, the thickness of that branch to the MN (d: 1.67 mm) was greater than the counterpart of the branch toward the CBM (d: 0.31 mm). This difference was indicative that the nerve to the CBM was not the main continuation of the MCN but only a motor branch.

**Fig. 2:**
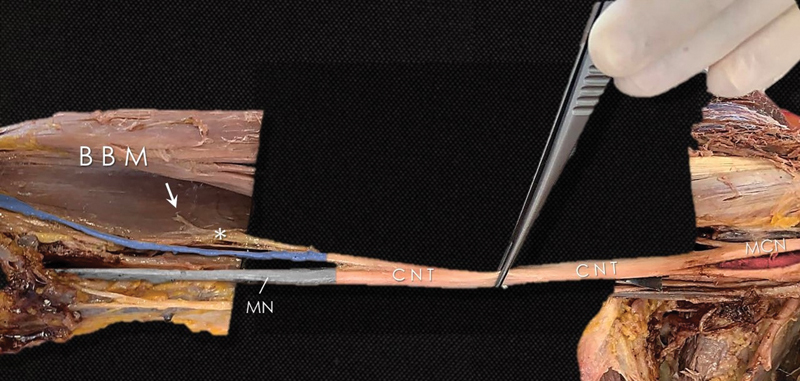
The division of the common neural trunk (CNT) into the median nerve (MN, *grey* ), muscular branches to the biceps brachialis muscle (BBM) and the lateral cutaneous antebrachii nerve ( *blue* ). MCN, musculocutaneous nerve.

Moreover, by continuing the dissection further ( Fig. 2 ), we identified some motor branches arising from the lateral side of the supposed to be MN toward the biceps brachii and a sensory neural branch that followed a course along the radial side of the forearm. So, this nerve was actually the lateral cutaneous antebrachii nerve and the rest of the neural stem continued as the MN.

**Fig. 3:**
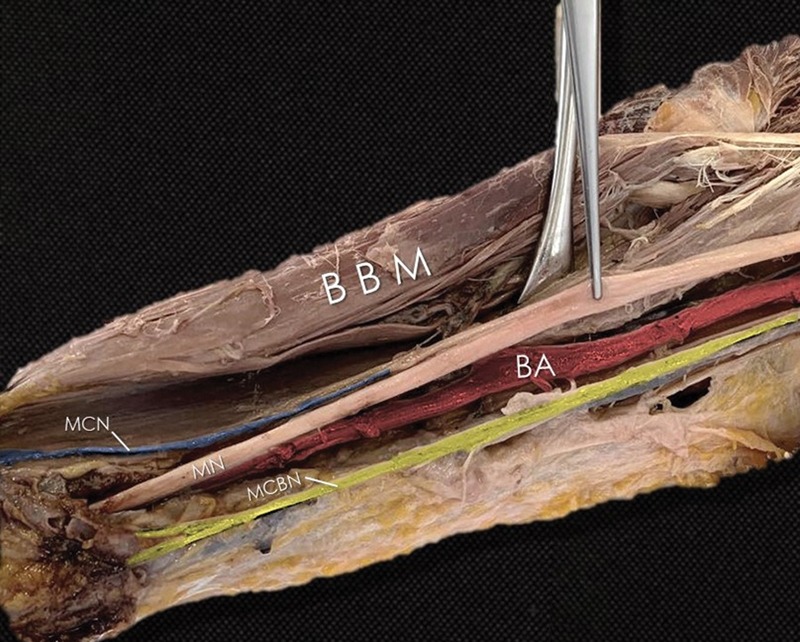
BA, brachial artery; BBM, biceps brachialis muscle; MCN, musculocutaneous nerve continuing as lateral cutaneous antebrachii nerve ( *blue* ); MCBN, medial cutaneous brachii nerve ( *yellow* ); MN, median nerve.

As a result, the case described was not a communication branch between the MCN and MN, or motor branches from the MN to the biceps brachii, but it was a fusion of the MN and MCN. After its origin from the lateral cord of the BP, the MCN gave off a branch to the CBM and then it merged with the MN. After covering some distance incorporated to a common neural trunk (CNT) (d: 2.96 mm), the two nerves finally separated and the MCN gave off some branches to the biceps brachii muscle and continued as the lateral cutaneous antebrachia nerve, while the MN continued its course distantly to the forearm and hand ( Fig. 3 ).

## Discussion

The embryological basis for the anatomical variations in the MCN and MN is dated to the 4th to 7th week of intrauterine development. Loops linking the neural fibers that innervate the limbs constitute a plexus. The anterior segmental branches are specifically combined to form the MN, which is followed by the MCN's emergence from the MN. 6 If the MCN does not get fully (or “appropriately”) separated from the MN, then the branching pattern of the two nerves may be altered. Usually, in these cases, a communicating neural branch between MCN and MN may occur or even a fusion between the two nerves as in the variation described.

By an extensive review of the relevant literature, we found only a few cases similar to this one reported. 7 8 It has been proposed that in such variations the MCN is hypoplastic and terminates its course into the CBM, and thus, the innervation of the rest anterior brachial muscles is aberrant from the MN. 7 However, we do believe (at least in our case) that the MCN is not terminated inside the CBM but it gives off a motor branch to the CBM and right after it incorporates to the MN. This perspective is also based on the definition of the MCN as provided by Guerri-Guttenberg and Ingolotti (2009) according to which the MCN is “ *the nerve that originates from the lateral cord of the brachial plexus at the point where the lateral root of the median nerve is detached from it* ” 9 . Indeed, in the reported case, the nerve that entered and innervated the CBM was not the one that detached from the MN's lateral root but only a branch from this nerve.

Other relatively common anatomical variations with the same embryological background involve the existence of communicating branches between the MN and MCN 10 and also the total absence of the MCN. In the last case, there is a full fusion of the MCN and MN as the MCN has never detached from the MN's lateral root, and thus all the three muscles of the anterior compartment of the arm are innervated by the MN. 11 12

Awareness of such anatomical variations and understanding of their background is of great significance for orthopaedic surgeons and neurosurgeons as they may complicate brachial plexus surgical procedures. 13 14 Also, aberrant nerves' formation and the existence of communicating neural branches may alter the clinical manifestation of nerve lesions (such as trauma, entrapment, and compression) and lead to misdiagnoses. 5 7 10
